# On Burden of Diseases, Prevention, Medical Research and Health Service Delivery: Grampian Case Study

**DOI:** 10.3390/ijerph23060763

**Published:** 2026-06-05

**Authors:** Seshadri S. Vasan, Sudarshan Anand, Miae Lee, Nicholas C. Fluck

**Affiliations:** 1 NHS Grampian, Summerfield House, Aberdeen AB15 6RE, UK; 2 School of Medical and Health Sciences, Edith Cowan University, Joondalup WA 6027, Australia; 3 School of Computational Science and Engineering, Georgia Institute of Technology, Atlanta, GA 30332, USA; 4 The Institute of Applied Health Sciences, University of Aberdeen, Aberdeen AB25 2ZD, UK

**Keywords:** burden of disease, compound annual growth rate (CAGR), disability-adjusted life years (DALYs), disease prevention, epidemiology, health policy, health service, health surveillance, medical research, public health

## Abstract

**Highlights:**

**Public health relevance—How does this work relate to a public health issue?**

- Disability-adjusted life years (DALYs) quantify population health loss across diseases and regions, while DALY rates enable meaningful comparisons between populations of different sizes and age structures.
- This study uses these measures to analyse regional disease burden and identify population health priorities in the Grampian region of Scotland.

**Public health significance—Why is this work of significance to public health?**

- The analysis identifies leading contributors to disease burden (e.g., cancers, ischaemic heart disease, Alzheimer’s disease) and highlights worsening trends in drug use disorders and colorectal cancer.
- The study demonstrates how publicly available surveillance data can be systematically analysed to generate actionable, region-specific insights beyond national averages, including variations by sex, age group, and sub-regions.

**Public health implications—What are the key implications or messages for practitioners, policy makers and/or researchers in public health?**

- Finding support targeted prevention strategies (e.g., early interventions for drug use disorders from adolescence and colorectal cancer screening from early adulthood), particularly in areas with higher DALY rates, such as Aberdeen City.
- The study provides a reproducible analytical framework to guide resource allocation, research prioritisation, and integrated public health and healthcare planning.

**Abstract:**

Burden of diseases measured as disability-adjusted life years (DALYs) per 100,000 people can be mined from public domain data, when they are made available by population health surveillance systems. This can be analysed to allow insightful comparisons with the national average, and to understand differences in trends between the sexes, age groups, time periods, geographic regions, and sub-regions. In this illustrative case study, we have analysed the Scottish burden of disease database to understand what ailed the population of the Grampian region before the COVID-19 pandemic. We have identified that selected cancers, ischaemic heart disease, Alzheimer’s disease and other dementias are amongst the highest contributors to the burden; that drug use disorders and colorectal cancer are showing worsening trends and require health promotion and disease prevention measures from ages 15 and 25, respectively, especially in Aberdeen City; and that males are more vulnerable to atrial fibrillation and flutter, diabetes mellitus, oesophageal cancer, and self-harm, while females are more vulnerable to cerebrovascular and chronic obstructive pulmonary diseases. We demonstrate the usefulness of our analysis and methodology for the wider health system, allowing targeted medical research investments and coordinated response from public health and health service delivery. We also show the need for up-to-date surveillance data, forecasts, and evidence on the impact of interventions to be made available widely.

## 1. Introduction

The origins of quality of life and cost-effectiveness analyses in healthcare can be traced back to a study on chronic renal disease published in 1968 [1], leading to quality-adjusted life years (QALYs) being formally defined in 1976 as the output of a utility function [2], c.f. Acknowledgements. This concept gained gradual acceptance over the next three decades for the economic evaluation of healthcare programmes [3], using metrics such as the incremental cost-effectiveness ratio developed at York [4], and the disability-adjusted life year (DALY) introduced in 1994 as a related term with age-weighting and discounting [5,6].

Disability-adjusted life year (DALY), the loss of equivalent of one year of full health, is a time-based measure that allows the burden of different diseases to be compared objectively. It is calculated as the sum of Years of Life Lost (YLL) due to premature mortality and Years Lived with Disability (YLD), i.e., DALY = YLL + YLD [5,6]. While DALYs quantify health loss at the population level, QALYs measure health gain by combining life expectancy with health-related quality of life [3]. In the United Kingdom (UK), QALYs are widely used in economic evaluation and health technology assessment by decision-making bodies such as the National Institute for Health and Care Excellence (NICE) to inform cost-effectiveness and resource allocation within the National Health Service (NHS) [7]. Thus, while QALYs support the evaluation of healthcare interventions, DALYs are particularly useful for identifying population health priorities.

DALY and DALY rate per 100,000 people in a given region can provide valuable insights to health service providers, public health departments, health economists, and policy makers if data are available to compare that region (for example, Grampian in Scotland) with others and the national average—as shown in this communication. Authentic and curated sources of such data in the UK include the Fingertips for England [8], the Scottish Burden of Disease [9], etc.

**Figure 1:**
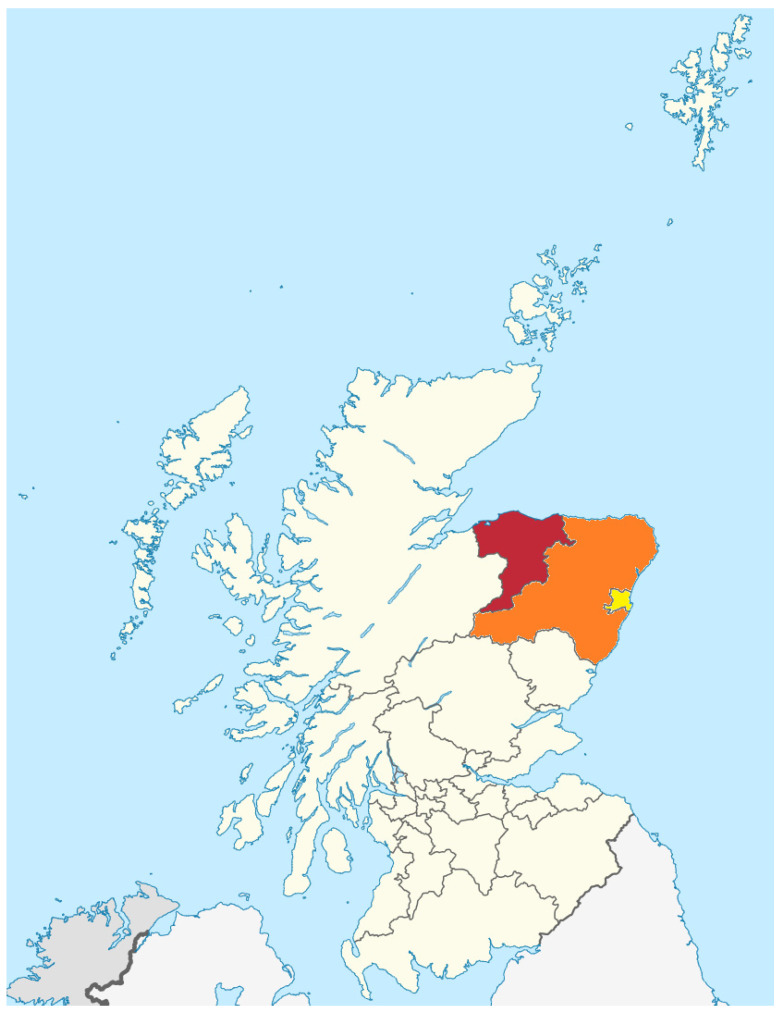
Grampian is a region in Scotland, and its constituent Health and Social Care Partnerships are shown in yellow (Aberdeen City), amber (Aberdeenshire), and red (Moray). Remixed image from Wikipedia released under the GNU Free Documentation License are highlighted for clarity.

Such burden of disease estimates are also useful for identifying sub-populations with poorer outcomes (within the scope of this paper), which in turn can lead to further studies (not in the scope of this paper) on the underlying socio-economic determinants of health for each sub-population, and likely interventions by public health and local authorities. The reason for this is as follows: the NHS Research and Development department, with data scientists, can often produce the former analysis (as we have done); however, investigation of the wider socio-economic determinants of health and implementation of appropriate interventions typically fall within the remit of the Health and Social Care Partnerships in Scotland, Integrated Care Systems in England, and related regional bodies. Analysis, such as the one we have done, can help inform the work of organisations, including Aberdeen City, Aberdeenshire, and Moray Health and Social Care Partnerships within NHS Grampian (Figure 1).

Grampian was selected as an illustrative case study due to its heterogeneous population, encompassing urban (including Aberdeen City), rural, and remote communities. The region also has a distinctive demographic profile influenced by global migration to the North Sea oil and gas sector. With circa 1% of the population of the UK, Grampian provides a representative setting to demonstrate how regional burden of disease data can be analysed to inform local health priorities. This communication is a descriptive study of regional burden of disease patterns intended to inform health policy, service planning, research prioritisation, and resource allocation through a reproducible analytical approach that can be applied by other health service providers worldwide.

## 2. Methods

For this work, we have used the Scottish Burden of Disease (SBoD) dataset [9], c.f. eight references therein, a population health surveillance system which monitors how diseases, injuries, and risk factors prevent the Scottish population from living longer lives in better health. DALY rates per 100,000 population were extracted for Scotland and the Grampian region. Morbidity estimates within the SBoD framework were derived from multiple sources, including linked primary care records, secondary care records, disease registers, national surveys, and communicable disease surveillance systems. Most estimates were based on routine administrative datasets linked using Community Health Index (CHI) numbers, while some conditions additionally incorporated self-reported (depression and anxiety) or aggregate survey data. The SBoD methodology estimates prevalent ill-health rather than incident diagnoses; however, the publicly available methodology does not specify a universal requirement for active treatment status or a fixed diagnostic ascertainment period across all conditions [9,10,11]. Although socio-economic stratification was not presented explicitly in this analysis, the underlying SBoD methodology incorporates age-specific, sex-specific, and Scottish Index of Multiple Deprivation (SIMD)-specific morbidity modelling, whereby national morbidity rates stratified by deprivation decile are applied to local population structures to estimate expected burden [10,12].

Analyses were conducted by sex, age group, and time period. Data were initially analysed for the years 2016 to 2019 [13]; 2014 and 2015 were included in this analysis when data were made available. In addition to cross-sectional comparisons for the most recent year available (2019), temporal trends were assessed using compound annual growth rates (CAGR), calculated as:

$$\mathrm{CAGR}=\left[{\left(\frac{Value\;at\;Year\;N}{Value\;at\;Year\;1}\right)}^{\frac{1}{N}}-1\right]\times 100$$

where *N* is the number of years. CAGR was calculated for the periods of 2014–19 and 2016–19 to capture both longer-term and more recent trends. Differences between CAGR estimates across time intervals reflect both temporal changes and sensitivity to baseline selection, particularly for conditions with smaller absolute annual changes or non-linear trends. The use of two time windows, therefore, provides complementary insights into disease trends.

Our analysis is descriptive in nature, focusing on identifying patterns and relative differences across regions and population subgroups. Formal statistical hypothesis testing was not undertaken because the publicly available SBoD dataset does not provide uncertainty intervals or sufficiently granular raw data for robust inferential statistical analyses. The SBoD methodology notes that conventional confidence intervals would not fully capture uncertainties related to modelling assumptions and disability weights [10]. Therefore, observed differences between regions and sub-regions should be interpreted cautiously as descriptive indicators of variation rather than statistically confirmed differences.

The underlying raw Scottish burden of disease data used by this communication is available as Supplementary Material Table S1.

## 3. Results and Discussions

Burden of disease expressed as DALY rates per 100,000 population was calculated for Scotland versus Grampian for the latest year for which data are available (2019), separately for females (Table 1 and Table 2) and males (Table 3 and Table 4).

It is seen from Table 1 and Table 3 that the leading causes of disease burden for Grampian are also important for Scotland; however, their exact order may vary. Ischaemic heart disease, lung cancer, Alzheimer’s disease, and other dementias significantly affect both Grampian and Scotland (c.f. Figure 1), so it is important to focus on these national priorities.

We are able to identify those diseases where Grampian’s DALY rate exceeds the Scottish average as local priorities for the region. These include atrial fibrillation and flutter, diabetes, and oesophageal cancer for males; breast cancer, cerebrovascular disease, and other cardiovascular and circulatory diseases for females; colorectal cancer and drug use disorders that affect both sexes with worsening trends since 2014 (c.f. Table 1 and Table 3).

Age-stratified analyses (Table 2 and Table 4) identify the age groups for these diseases of concern where the burden is especially high. For example, Alzheimer’s disease and other dementias primarily affect those aged 65+, whereas drug use disorders and self-harm disproportionately impact younger and working-age populations. We assessed temporal trends using CAGR for 2014–19 as well as 2016–19 (as previously computed [13]) to identify diseases with significant temporal changes that warrant closer monitoring in future analyses. Application of CAGR across two time intervals (2014–2019 and 2016–2019) enabled the identification of additional priority conditions.

It is important to address these unmet needs through a combination of public health measures (e.g., health promotion, disease prevention) and interventions arising from life sciences, health, and medical research and innovation [14]. The latter is very important as demonstrated by, for example, the recent reviews of Australia’s Medical Research Future Fund [15,16], in which it was found that 231 grants were awarded during 2016–19 with a total value of AU$574.5 million [16], but when mapped against 17 disease groups in the Australian burden of disease study 2015 [17], only a weak association was observed with DALY (*r*^2^ = 0.4359) and no association was observed with disability burden YLD (*r*^2^ = 0.0009) [16]. Any life sciences, health, and medical research and innovation investments into Grampian by public, private, or non-profit sectors should take these considerations into account. Realistic medicine approaches [18] and regional investments should target the local burden of diseases, lest there could be higher opportunity costs and unintentional widening of inequalities. For the Grampian region, disease priorities are summarised under the mnemonic “CICADAS” (Table 5), with the worst-affected age groups identified from Table 2 and Table 4.

As shown in Figure 1 and previously described, the Grampian region is in turn comprising three Health and Social Care Partnerships (viz. Aberdeen City, Aberdeenshire, and Moray), which are local authorities in Scotland responsible for planning and delivering health and social care services in partnership with the NHS. Examining sub-regional variations can provide valuable insights for these partnerships to identify relevant socio-economic determinants of health and devise appropriate interventions for each affected sub-population. For example, the Aberdeen City Health and Social Care Partnerships is one of the UK’s ‘Marmot Places’, an initiative focused on reducing health inequalities through action on the social determinants of health [19].

From Table 6, it is seen that Aberdeenshire is close to the Grampian average for diseases in females listed under Table 2; Aberdeen City has a higher DALY rate than Grampian for Alzheimer’s disease and other dementias, lung and colorectal cancers, and drug use disorders; while Moray has a higher DALY rate than Grampian for ischaemic heart disease, cerebrovascular disease, and other cardiovascular and circulatory diseases.

From Table 7, it is seen that Aberdeenshire is below the Grampian average for diseases in males listed under Table 4 (except perhaps colorectal cancer, atrial fibrillation, and flutter); Moray has a higher DALY rate than Grampian for lung cancer, atrial fibrillation, and flutter; while Aberdeen has a higher DALY rate than Grampian for all but atrial fibrillation and flutter. Thus, we notice that while some trends are the same for both sexes, there are significant differences too, so we need a nuanced approach. Observed regional variations may partly reflect underlying socio-economic differences between urban, rural, and mixed populations across the Grampian region, including deprivation, occupational structure, environmental exposures, and differential access to healthcare and diagnostic services. In addition to these factors, broader contextual influences such as industrial activity, air quality, and natural environmental characteristics may also contribute to differences in disease burden, although these were not directly measured in this study.

## 4. Conclusions, Limitations, and Future Work

With health services under increasing pressure across the world, it is important to ensure better alignment between the long-term plans for population health and integrated health and social care. In many developed countries, we have health surveillance data in the public domain. These, especially on the burden of diseases, can be mined and analysed by health service providers to serve their populations more effectively and in a targeted manner, as shown in this communication with the Grampian region case study. For example, we were able to identify which diseases have the highest DALY rate burden, which ones are of particular concern to Grampian, and those that are showing a worsening trend. We were also able to gain a nuanced understanding in terms of differences between males and females, age groups, and the three sub-regions that make up Grampian. This will allow targeted medical research investments and coordinated response from public health and health service delivery [14].

Importantly, while DALYs are well-suited for population health assessment and prioritisation, decision-making within the UK’s National Health Service is typically informed by cost-effectiveness frameworks based on QALYs, particularly in health interventions [7]. Although QALY-based analyses are beyond the scope of this study, our findings provide robust, region-specific burden of disease data that can inform policy and resource allocation. These quantitative results can be complemented by qualitative analyses, such as the recent Grampian participatory research on patient, public, and stakeholder perspectives, which identifies priority health areas from the viewpoint of those directly affected [14]. Together, such quantitative and qualitative evidence can strengthen local NHS planning, providing a foundation that can be expanded to inform broader decision-making in epidemiology and health policy.

We acknowledge that the public domain Scottish burden of disease data extends only to 2019, limiting direct applicability to post-pandemic health contexts. However, this communication does provide a useful pre-pandemic baseline for future comparisons. The methodological framework remains robust and can be readily applied to updated datasets as they become available.

Future work in Scotland, perhaps led by the relevant Health and Social Care Partnerships, should incorporate socio-economic stratification using measures such as SIMD deciles to enable a more detailed assessment of health inequalities within and between sub-regions, thereby supporting more targeted and equitable health planning. Finer-scale analyses within regions, alongside consideration of environmental, occupational, and healthcare access factors, may further help to explain observed variations in disease burden across sub-regions and what targeted interventions are needed.

There are several categories of public domain SBoD data published by Public Health Scotland, which researchers are unable to modify and therefore act as limiting factors for our analysis. For example, self-harm and interpersonal violence are distinct with very dissimilar causes, social outcomes, policies, and prevention programmes; however, these are only available to us as an aggregated line item. Several categories, such as “other cardiovascular” or “other cancers”, are too broad and less specific in the underlying dataset, highlighting the need for more granular categorisation in the public domain.

Crucially, this study provides a template that other regions can adapt using their own local data. It highlights the need for future analyses incorporating improved data granularity and socio-economic stratification that would enable more targeted and equitable health planning, and provide a more nuanced understanding of regional health inequalities. We further demonstrate the importance of up-to-date surveillance data being available to health service providers, as well as the need for predictive trends and evidence on the impact of interventions, and it is heartening to note that such forecasting could soon be made available “to offer insights into future public health challenges in Scotland” [9].

## Figures and Tables

**Table 1:** Disability-adjusted life year (DALY) rate per 100,000 people—females in Scotland versus Grampian (across time).

FEMALES										
Disease	Scotland	Grampian								
	2019	2014	2015	2016	2017	2018	2019	Difference ^6^	CAGR ^7^ 2014–2019 (%)	CAGR ^7^ 2016–2019 (%)
**Alzheimer’s disease** ^1^	1827	1472	1636	1616	1655	1675	**1693**	134	**2.84**	**1.56**
**Ischaemic heart disease**	1605	1543	1402	1525	1639	1635	**1489**	116	−0.71	−0.79
**Lung cancer**	1474	1355	1337	1163	1110	1287	**1366**	108	0.16	**5.51**
Low back and neck pain	1452	1370	1370	1370	1391	1391	1390	62	0.29	0.48
**Cerebrovascular disease**	1379	1488	1510	1508	1521	1391	1414	**−35**	−1.02	−2.12
Headache disorders	1333	1328	1327	1327	1337	1337	1337	−4	0.14	0.25
**COPD** ^2^	1301	992	1252	1060	1144	952	1087	214	**1.85**	0.84
Depression	1214	1045	1045	1044	1068	1068	1067	147	0.42	0.73
**Other cancers**	1094	985	1061	930	1153	1169	911	183	−1.55	−0.69
Anxiety disorders	1045	899	899	898	919	919	918	127	0.42	0.74
**Breast cancer**	1027	1328	1160	1106	1092	846	1077	**−50**	−4.1	−0.88
**Drug use disorders**	1003	311	401	553	683	522	677	326	**16.83**	**6.98**
**Other cardiovascular** ^3^	763	1096	924	837	865	679	850	**−87**	−4.96	0.52
**Colorectal cancer**	648	690	715	638	681	668	726	**−78**	**1.02**	**4.40**
Lower respiratory infections	622	526	690	612	800	691	528	94	0.08	−4.80
Diabetes mellitus	586	513	513	555	600	551	487	99	−1.03	−4.26
Cirrhosis ^4^	510	454	434	445	396	429	421	89	−1.5	−1.83
**Other musculoskeletal disorders**	462	451	463	466	475	478	445	17	−0.27	−1.53
Osteoarthritis	457	438	438	438	442	442	442	15	0.18	0.30
Other digestive diseases	440	371	357	387	378	344	368	72	−0.16	−1.66
**Skin and subcutaneous diseases**	406	399	400	364	386	406	391	15	−0.40	**2.41**
**Self-harm** ^5^	399	282	449	289	311	291	332	67	**3.32**	**4.73**
**Chronic kidney disease**	382	399	420	376	499	423	396	−14	−0.15	**1.74**
**Falls**	380	366	299	277	316	287	332	48	−1.93	**6.22**
Gynaecological diseases	368	358	358	367	360	360	360	8	0.11	−0.64
Asthma	330	320	383	350	392	317	334	−4	0.86	−1.55

^1^ and other dementias; ^2^ chronic obstructive pulmonary disease; ^3^ and circulatory diseases; ^4^ and other chronic liver diseases; ^5^ and interpersonal violence; ^6^ difference between 2019 DALY rates for Scotland minus Grampian; ^7^ compound annual growth rate in percentage. **Bold font** indicates areas of concern.

**Table 2:** Disability-adjusted life year (DALY) rate per 100,000 people—females in Grampian 2019 (for selected diseases, age groups).

FEMALES							
Selected Diseases of Concern	Grampian 2019						
	All Ages	Under 15	15 to 24	25 to 44	45 to 64	65 to 84	85 and Over
Alzheimer’s disease ^1^	1693	0	0	2	72	**4489**	**36,399**
Ischaemic heart disease	1489	0	1	81	**1012**	**4929**	**14,448**
Cerebrovascular disease	1414	186	8	113	**913**	**3878**	**18,098**
Lung cancer	1366	0	0	73	**1765**	**4778**	**2660**
COPD	1087	0	15	133	**865**	**4143**	**4646**
Breast cancer	1077	0	18	**742**	**1774**	**1926**	**3218**
Other cancers	911	2	4	**535**	**1201**	**2174**	**3216**
Other cardiovascular ^2^	850	26	37	**300**	**872**	**2250**	**5948**
Colorectal cancer	726	5	7	**383**	**530**	**2271**	**3851**
Drug use disorders	677	0	**430**	**1763**	**561**	65	8
Other musculoskeletal disorders	445	84	271	**388**	**530**	**680**	**1667**
Chronic Kidney Disease	396	0	2	8	227	**1266**	**4738**
Skin and subcutaneous diseases	391	**435**	**539**	**343**	243	**500**	**773**
Falls	332	58	71	40	99	**792**	**5706**
Self-harm ^3^	332	1	**1039**	**381**	**365**	68	118

^1^ and other dementias; ^2^ circulatory diseases; and ^3^ interpersonal violence. **Bold font** indicates areas of concern.

**Table 3:** Disability-adjusted life year (DALY) rate per 100,000 people—males in Scotland versus Grampian (across time).

MALES										
Cause	Scotland	Grampian								
	2019	2014	2015	2016	2017	2018	2019	Difference ^6^	CAGR ^7^ 2014–2019 (%)	CAGR ^7^ 2016–2019 (%)
**Ischaemic heart disease**	3713	3909	3353	3311	3495	3422	**3488**	225	−2.25	**1.75**
**Drug use disorders**	2367	889	887	1007	1337	1308	**1233**	1134	**6.76**	**6.98**
**Lung cancer**	1749	1731	1656	1842	1737	1738	**1605**	144	−1.5	−4.49
**Alzheimer’s disease ^1^**	1639	1504	1598	1370	1536	1578	1592	47	**1.14**	5.13
Cerebrovascular disease	1516	1629	1776	1800	1720	1763	1291	225	−4.54	−10.49
COPD ^2^	1296	1279	1456	1208	1385	1093	1208	88	−1.14	0
**Other cancers**	1295	1004	1221	1259	1235	1108	1142	153	**2.61**	−3.2
Depression	1277	1058	1059	1059	1092	1091	1090	187	0.6	0.97
**Self-harm ^3^**	1194	871	1010	930	895	972	934	260	**1.41**	0.14
Other cardiovascular ^4^	1077	1402	1242	1219	1241	1146	924	153	−8	−8.82
Low back and neck pain	1075	1029	1029	1028	1041	1041	1040	35	0.21	0.39
**Diabetes mellitus**	923	759	736	816	924	935	973	**−50**	**5.09**	**6.04**
**Colorectal cancer**	878	780	833	808	1012	1083	960	**−82**	**4.24**	**5.91**
Alcohol use disorders	863	583	704	637	720	714	607	256	0.81	−1.6
Prostate cancer	821	926	1043	773	830	761	758	63	−3.92	−0.65
**Lower respiratory infections**	801	844	850	671	884	736	774	27	−1.72	**4.88**
Cirrhosis ^5^	752	556	650	747	592	596	496	256	−2.26	−12.76
Anxiety disorders	646	536	536	536	553	552	552	94	0.59	0.99
Headache disorders	617	619	619	619	623	623	623	−6	0.13	0.21
**Oesophageal cancer**	483	596	727	491	545	498	617	**−134**	0.69	**7.91**
**Falls**	466	336	324	455	353	424	366	100	**1.73**	−7
**Atrial fibrillation and flutter**	430	382	434	447	493	412	454	**−24**	**3.51**	0.52
**Other digestive diseases**	412	331	416	376	327	351	368	44	**2.14**	−0.71
**Other chronic respiratory diseases**	395	341	338	323	353	428	360	35	**1.09**	**3.68**
Chronic kidney disease	394	439	368	394	389	385	388	6	−2.44	−0.51
**Other musculoskeletal disorders**	385	379	416	374	356	379	387	−2	0.42	**1.15**
**Pancreatic cancer**	372	311	309	285	382	436	366	6	**3.31**	**8.7**

^1^ and other dementias; ^2^ chronic obstructive pulmonary disease; ^3^ and interpersonal violence; ^4^ and circulatory diseases; ^5^ and other chronic liver diseases; ^6^ difference between 2019 DALY rates for Scotland minus Grampian; ^7^ compound annual growth rate in percentage. **Bold font** indicates areas of concern.

**Table 4:** Disability-adjusted life year (DALY) rate per 100,000 people—males in Grampian 2019 (for selected diseases, age groups).

MALES							
Cause	Grampian 2019						
	All Ages	Under 15	15 to 24	25 to 44	45 to 64	65 to 84	85 and Over
Ischaemic heart disease	3488	1	2	**391**	**3577**	**11,196**	**21,317**
Lung cancer	1605	0	0	**141**	**1574**	**5874**	**6083**
Alzheimer’s disease ^1^	1592	0	0	1	204	**4632**	**29,994**
Drug use disorders	1233	0	**884**	**2671**	**1564**	21	193
Other cancers	1142	174	248	**364**	**1349**	**3209**	**3429**
Diabetes mellitus	973	19	62	**286**	**1266**	**2802**	**3015**
Colorectal cancer	960	6	11	94	**872**	**3327**	**5437**
Self-harm ^2^	934	2	**1161**	**1690**	**970**	**442**	**796**
Oesophageal cancer	617	0	1	70	**734**	**2235**	**954**
Atrial fibrillation and flutter	454	0	3	20	**229**	**1601**	**4630**
Other musculoskeletal disorders	387	91	230	**314**	**520**	**612**	**832**
Pancreatic cancer	366	0	0	0	**611**	**999**	**1363**
Falls	366	74	125	93	**204**	**820**	**4851**
Other chronic respiratory diseases	360	1	4	8	**227**	**1343**	**2746**

^1^ and other dementias; ^2^ and interpersonal violence. **Bold font** indicates areas of concern.

**Table 5:** Selected disease conditions of concern to Grampian and the age groups worst affected.

Acronym	Disease Condition(s) of Concern	Age Groups Worst Affected
C	**C**ancer–breast, colorectal, lung, oesophageal (especially amongst males)	25+
I	**I**schaemic heart disease; other cardiovascular and circulatory diseases	25+
C	**C**erebrovascular disease and COPD (especially amongst females)	45+
A	**A**lzheimer’s disease and other dementias	65+
D	**D**rug use disorders	15–64
A	**A**trial fibrillation and flutter (especially amongst males)	45+
S	**S**pecific conditions listed below (ordered by age groups)	
	Self-harm and interpersonal violence (especially amongst females) ^1^ Diabetes mellitus (especially amongst males) Other musculoskeletal disorders Lower respiratory infections (especially amongst males) Other chronic respiratory diseases (especially amongst males) Other digestive diseases (especially amongst males)	15–64 25+ 25+ 45+ 65+ 65+

^1^ Self-harm and interpersonal violence are combined as defined in the SBoD dataset and may represent conditions with differing underlying determinants and contextual factors.

**Table 6:** Disability-adjusted life year (DALY) rate per 100,000 people—females (2019, for selected diseases in Table 2, sub-regions).

Selected Diseases of Concern	Grampian	Aberdeen	Aberdeenshire	Moray
Alzheimer’s disease ^1^	1693	**1821**	1660	1526
Ischaemic heart disease	1489	1460	1477	**1597**
Cerebrovascular disease	1414	**1493**	1252	**1683**
Lung cancer	1366	**1603**	1207	1334
Breast cancer	1077	1068	**1113**	1012
Other cardiovascular ^2^	850	797	850	**874**
Colorectal cancer	726	**960**	674	438
Drug use disorders	677	**830**	638	449

^1^ and other dementias; ^2^ and circulatory diseases. **Bold font** indicates areas of concern. Values are presented descriptively to illustrate regional variation. No formal statistical comparisons were conducted, and differences should be interpreted cautiously.

**Table 7:** Disability-adjusted life year (DALY) rate per 100,000 people—males (2019, for selected diseases in Table 4, sub-regions).

Selected Diseases of Concern	Grampian	Aberdeen	Aberdeenshire	Moray
Ischaemic heart disease	3488	**4166**	3109	3231
Lung cancer	1605	**1910**	1287	**1898**
Alzheimer’s disease ^1^	1592	**1732**	1556	1440
Drug use disorders	1233	**1665**	949	1153
Diabetes mellitus	973	**1153**	869	930
Colorectal cancer	960	**1103**	**980**	682
Oesophageal cancer	617	**823**	538	449
Atrial fibrillation and flutter	454	399	**478**	**503**

^1^ and other dementias. **Bold font** indicates areas of concern. Values are presented descriptively to illustrate regional variation. No formal statistical comparisons were conducted, and differences should be interpreted cautiously.

## Data Availability

Data supporting reported results can be downloaded from the Scottish burden of disease [9] or the Supplementary Materials.

## Supplementary Materials

The following supporting information can be downloaded at: https://www.mdpi.com/article/10.3390/ijerph23060763/s1, Table S1: Scottish burden of disease underlying dataset.
